# Sjogren-Larsson syndrome brain volumetric reductions demonstrated with an automated software

**DOI:** 10.1055/s-0043-1772601

**Published:** 2023-10-04

**Authors:** José Thiago de Souza de Castro, Camilo Lotfi Saab, Mariam Patrícia Auada Souto, Juliane Giselle Ortolam, Carlos Eduardo Steiner, Thiago Junqueira Ribeiro de Rezende, Fabiano Reis

**Affiliations:** 1 Universidade Estadual de Campinas, Faculdade de Ciências Médicas, Departamento de Anestesiologia, Oncologia e Radiologia, Campinas SP, Brazil.; 2 Universidade Estadual de Campinas, Faculdade de Ciências Médicas, Departamento de Clínica Médica, Campinas SP, Brazil.; 3 Universidade Estadual de Campinas, Faculdade de Ciências Médicas, Departamento de Medicina Translacional , Campinas SP, Brazil.; 4 Universidade Estadual de Campinas, Faculdade de Ciências Médicas, Departamento de Neurologia, Campinas SP, Brazil.

**Keywords:** Sjogren-Larsson Syndrome, Neurocutaneous Syndromes, Thalamic Diseases, Brain Diseases, Síndrome de Sjogren-Larsson, Síndromes Neurocutâneas, Doenças Talâmicas, Encefalopatias

## Abstract

**Background** Sjogren-Larsson syndrome (SLS) is a neurocutaneous disease with an autosomal recessive inheritance, caused by mutations in the gene that encodes fatty aldehyde dehydrogenase ( *ALDH3A2* ), clinically characterized by ichthyosis, spastic diplegia, and cognitive impairment. Brain imaging plays an essential role in the diagnosis, demonstrating a nonspecific leukoencephalopathy. Data regarding brain atrophy and grey matter involvement is scarce and discordant.

**Objective** We performed a volumetric analysis of the brain of two siblings with SLS with the aim of detecting deep grey matter nuclei, cerebellar grey matter, and brainstem volume reduction in these patients.

**Methods** Volume data obtained from the brain magnetic resonance imaging (MRI) of the two patients using an automated segmentation software (Freesurfer) was compared with the volumes of a healthy control group.

**Results** Statistically significant volume reduction was found in the cerebellum cortex, the brainstem, the thalamus, and the pallidum nuclei.

**Conclusion** Volume reduction in grey matter leads to the hypothesis that SLS is not a pure leukoencephalopathy. Grey matter structures affected in the present study suggest a dysfunction more prominent in the thalamic motor pathways.

## Resumo

**Antecedentes** A Síndrome de Sjogren-Larsson (SSL) é uma doença neurocutânea de herança autossômica recessiva, causada por mutações no gene que codifica a aldeído graxo desidrogenase ( *ALDH3A2* ), caracterizada clinicamente por ictiose, diplegia espástica e comprometimento cognitivo. A imagiologia cerebral desempenha um papel essencial no diagnóstico, demonstrando uma leucoencefalopatia inespecífica. Dados sobre atrofia cerebral e envolvimento da substância cinzenta são escassos e discordantes.

**Objetivo** Realizamos uma análise volumétrica do cérebro de dois irmãos com SLS com o objetivo de detectar núcleos profundos de substância cinzenta, substância cerebral cinzenta e redução do volume do tronco encefálico nestes pacientes.

**Métodos** Os dados de volume obtidos da ressonância magnética (RM) cerebral dos dois pacientes usando um software de segmentação automática (Freesurfer) foram comparados com os volumes de um grupo controle saudável.

**Resultados** Redução de volume estatisticamente significativa foi encontrada no córtex do cerebelo, no tronco cerebral, no tálamo e nos núcleos pálidos.

**Conclusão** A redução do volume da substância cinzenta leva à hipótese de que a SSL não é uma leucoencefalopatia pura. As estruturas da substância cinzenta afetadas no presente estudo sugerem uma disfunção mais proeminente nas vias motoras talâmicas.

## INTRODUCTION

Sjogren-Larsson syndrome (SLS) is a neurocutaneous disease with an autosomal recessive inheritance, caused by mutations in the gene that encodes fatty aldehyde dehydrogenase ( *ALDH3A2* ). It affects predominantly the skin and brain, resulting in the classic triad of ichthyosis, spastic diplegia, and cognitive impairment. 1 2 Brain imaging plays an essential role in the diagnosis, albeit with nonspecific findings, the most prominent of which is a leukoencephalopathy. Previous data from brain magnetic resonance imaging (MRI) of SLS patients also report brain atrophy in some individuals. 3 4 While white matter involvement is well established, data related to grey matter involvement is scarce, with some neuropathological reports indicating discordant areas of affected grey matter. In the present study, we performed a volumetric analysis of the brain MRI of two siblings with SLS using an automated segmentation software (Freesurfer) and compared the volumes obtained to the data of a healthy control group, with the aim of detecting grey and/or white matter volume reduction in these patients.

## METHODS

### Patients

Patients that had a confirmed diagnosis of SLS and who were followed in our institution were selected for the case group. Patients with presumptive diagnosis and no confirmatory test were excluded from the selection.

For the control group, we searched for subjects with no medical background in our brain MRI database and selected them on the basis of sex and age so as to match the case group. Subjects with structural alterations in the brain were excluded from the study.

The Institutional Review Board/Ethics Committee, Research Ethics Committee of the Faculty of Medical Sciences – Universidade de Campinas (UNICAMP, in the Portuguese acronym) approved the study, and informed consent was obtained for all participants.

### Magnetic resonance imaging

Magnetic resonance imaging was performed on all patients and control subjects on a high resolution 3T Achieva Intera Scanner (Philips, Amsterdam, Netherlands) using a standard protocol consisting of routine T1 and T2 weighted sequences. Additionally, for the FreeSurfer analysis, a volumetric T1-weighted sequence was acquired using a standard 8 channel head coil, with a sagittal orientation, voxel matrix 240 × 240 × 180, voxel size 1 × 1 × 1mm3, TR/TE = 7.0/3.2 ms, flip angle 8°.

### Volumetry

Cortical and subcortical segmentation and volumetric analysis was performed with the Freesurfer 6.0 image analysis suite, which is available for download online ( http://surfer.nmr.mgh.harvard.edu/ ). Technical details can be found in prior publications. 5 6 7 8 Briefly, in its image processing, it includes field inhomogeneity correction, skull removal, and alignment to Talaraich atlas, followed by the labelling of each voxel as grey matter, white matter, or cerebrospinal fluid and tessellation of the grey matter/white matter boundaries. The maps created rely on both the absolute signal intensity and spatial intensity gradients across the tissues and provide sub millimeter resolution, beyond the voxel resolution. The process was checked for errors and the need of manual adjustments, although no correction was warranted. We included in our analysis the volumes of the deep grey matter structures (caudate, putamen, and pallidum nuclei, as well as the thalamus), the brainstem, and the cerebellar cortex. Due to the cerebral white matter signal alteration that is inherent to the disease, we did not include neither supratentorial cortical thickness and volume nor cerebral white matter volume in the analysis considering the possibility that the software could misinterpret the cortical grey matter/white matter boundaries.

### Statistical analysis

The normality of the distribution of the volumes obtained from the control group was tested using the Shapiro-Wilk test, confirming a normal distribution with *p-values* > 0.05. The brain bilateral structures (cerebellar cortex, thalamus, pallidum, caudate and putamen) had their volumes represented by the average of the volumes of both hemispheres. The mean volumes of the case group were compared with the control group using a one-sample Z-test, since we disposed of the population (control group) standard deviation (SD) and variance values. The test was one-tailed, considering that the previous literature data indicated only brain volume reductions in SJL patients. The primary significance level was *p* < 0.05, adjusted to *p* < 0.008 after the Bonferroni correction for multiple comparisons, to avoid type I errors.

## RESULTS

### Participants

The search in our institution database returned three patients that fulfilled the criteria for the case group. They were siblings from non-consanguineous parents from a small, isolated community where consanguinity is more prevalent and a founder effect for the SLS could be identified. One of them had to be excluded from the analysis since the software used could not conclude brain segmentation due to profuse white matter signal alteration. The patients included in our study were females aged 22 and 37 years old (mean age: 29.5 years old) at the time of investigation. Both of them presented with the classical triad of congenital ichthyosis, spasticity, and mental retardation in the first years of life, and had their diagnosis confirmed by molecular characterization of the *ALDH3A2* gene mutation (both had a homozygous mutation c.1108-1G > C in intron 7). They had moderate cognitive impairment and were dependent on wheelchair.

We included in the control group 41 healthy patients, all females, with ages similar to the case group (mean age 27.9 years old; range 20 to 37 years old). None had structural alterations in the brain.

### MRI analysis

Both SLS patients presented symmetrical, confluent T2/FLAIR hyperintensity in the periventricular white matter, predominantly in the frontal lobes, sparing subcortical fibers. The older patient also presented T1 hypointensity in these regions.

### Data analysis

All volumes obtained from the control group were found to be normally distributed using the Shapiro-Wilk test.

**Figure 1:**
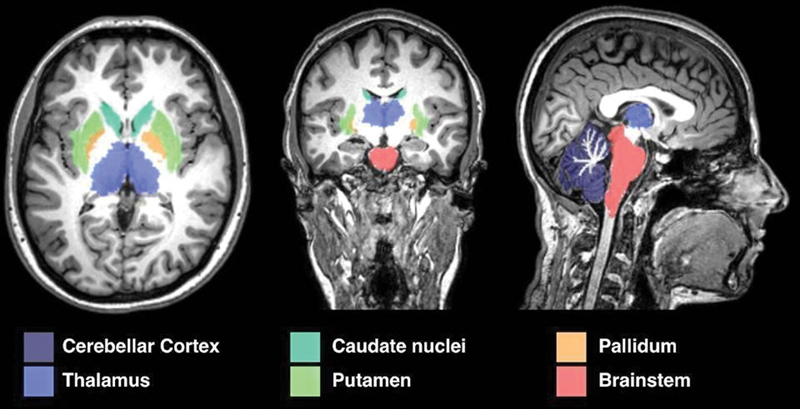
Schematic view of the analyzed structures segmentation.

**Figure 2:**
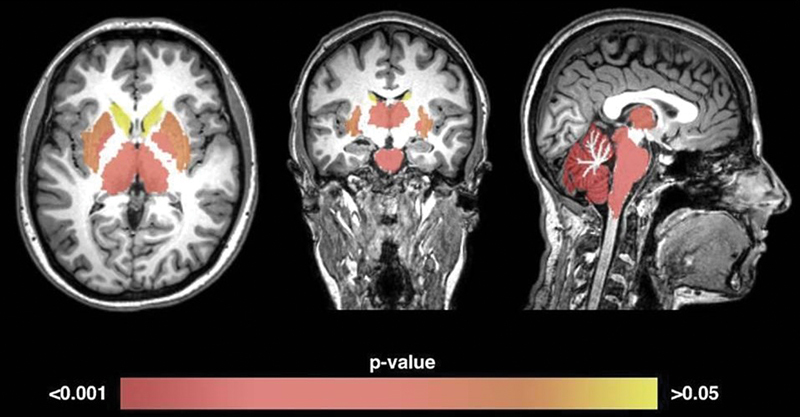
P-values obtained from each structure represented in a color scale. Structures without statistically significant volume reduction are represented in orange (putamen: *p* = 0.01) and yellow (caudate nuclei: *p* = 0.3).

**Figure 3:**
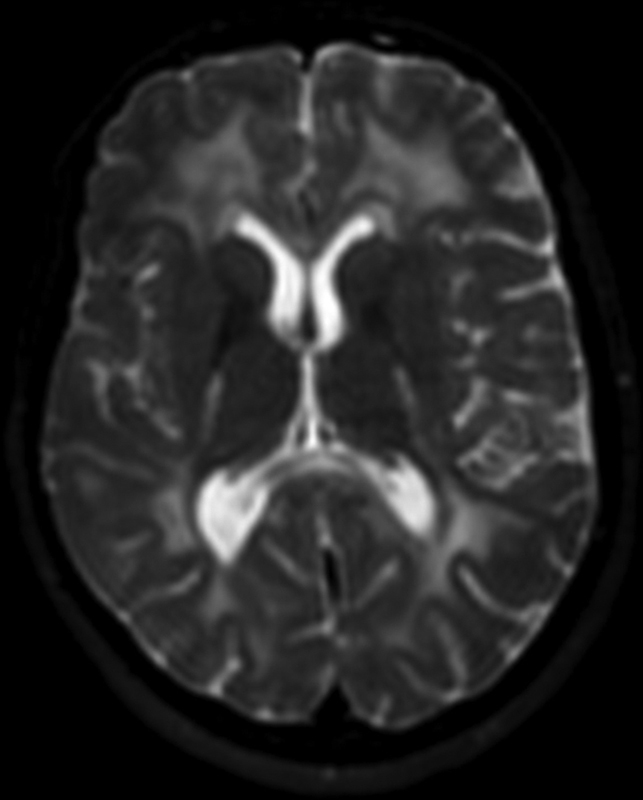
Axial T2 weighted image demonstrates confluent and symmetrical cerebral white matter hyperintensities in the periventricular regions and in the posterior limb of the internal capsules.

**Figure 4:**
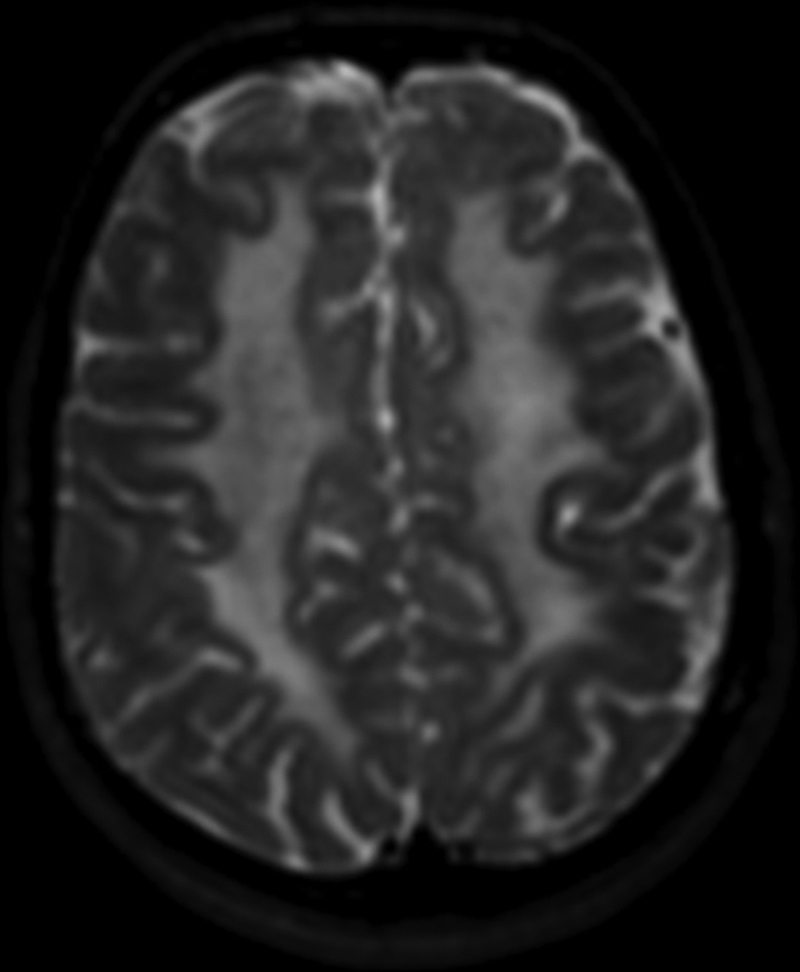
Axial T2 weighted image at the level of the centrum semiovale demonstrates confluent and symmetrical deep white matter hyperintensities.

The structures analyzed are represented on Figure 1 . There was a significant volume reduction of the cerebellum cortex ( *p* < 0.001), the brainstem ( *p* = 0.002), the thalami ( *p* = 0.005), and the pallidum nuclei ( *p* = 0.005) ( Figure 2 ). We did not find significant reduction in the volume of the caudate nuclei ( *p* = 0.3) and putamen ( *p* = 0.01). The Table 1 demonstrates neuroimaging data with FreeSurfer. The severity of white matter disease is demonstrated in Figures 3 and 4 .

**Table 1:** Volumes (mean ± SD) obtained using FreeSurfer, in cubic centimeters.

Volumes	Control group	SLS patients	Z-score	*p-value*
Total brain	1071.52 ± 65.82	968.79 ± 10.14	- 2.20	0.013
Cerebral WM	425.70 ± 39.10	329.18 ± 41.07	- 3.49	< 0.001
Cortex	444.43 ± 26.37	428.13 ± 11.61	- 0.87	0.192
GM	601.13 ± 32.50	561.95 ± 5.16	- 1.70	0.044
Brainstem	19.55 ± 1.92	15.74 ± 0,14	- 2.79	<0.002
Left caudate	3.52 ± 0.37	3.56 ± 0.03	0.15	0.440
Right caudate	3.63 ± 0.36	3.47 ± 0.01	- 0.62	0.264
Left cerebellum cortex	49.48 ± 3.45	40.51 ± 2.43	- 3.67	<0.001
Right cerebellum cortex	49.61 ± 3.39	41.50 ± 2.60	- 3.37	<0.001
Left cerebellum WM	14.41 ± 1.42	11.53 ± 0.41	- 2.85	<0.003
Right cerebellum WM	13.59 ± 1.40	11.09 ± 1.01	- 2.52	<0.006
Left hippocampus	4.03 ± 0.30	3.95 ± 0.15	- 0.37	0.351
Right hippocampus	4.14 ± 0.32	3.79 ± 0.00	- 1.56	0.058
Left pallidum	1.86 ± 0.18	1.51 ± 0.19	- 2.85	<0.002
Right pallidum	1.84 ± 0.16	1.56 ± 0.17	- 2.80	<0.003
Left putamen	4.98 ± 0.39	4.45 ± 0.66	- 1.93	0.026
Right putamen	4.96 ± 0.38	4.37 ± 0.55	- 2.22	0.012
Left thalamus	7.14 ± 0.66	5.92 ± 0.44	- 2.60	<0.005
Right thalamus	6.77 ± 0.55	5.74 ± 0.07	- 2.61	<0.005

Abbreviations: SD, standard deviation; SLS, Sjogren-Larsson syndrome; WM, white matter; GM, grey matter. Note: *significant *p* < 0.008 (Bonferroni corrected one-tailed Z-test).

## DISCUSSION

Sjogren-Larsson syndrome is an inherited autosomal recessive neurocutaneous disease caused by an inborn error of the lipid metabolism resulting from mutations in the *ALDH3A2* gene that encodes fatty aldehyde dehydrogenase (FALDH). Fatty aldehyde dehydrogenase is an enzyme involved, among other lipid metabolism pathways, in the removal of aldehydes from both endogenous and exogenous sources through oxidation into fatty acids. Its activity deficiency leads to abnormal lipid accumulation in the membranes of the skin and brain. In the skin, it leads to disruption of the epidermal water barrier and transepidermal water loss, while in the brain it may alter the structure and/or the function of myelin membranes. 1 2 4 9 10 11 There are several mutations that result in SLS, some being more common in certain geographical distributions. The c.1108-1G > C variant was initially described in the Iberian Peninsula and is probably the most common pathogenic mutation in Brazilian patients with SLS. 1

Clinically, it is characterized by a classic triad of ichthyosis, spastic diplegia, and cognitive impairment. The ichthyosis is typically congenital in onset with erythema and scaling apparent in the neonatal period. 12

Neurologic symptoms become evident in the first or second year of life, with a delay in motor milestones as a consequence of spasticity. This motor disability tends to be more prominent in legs, leading to difficulty in ambulation, uncommonly affecting upper limbs. The majority of patients with SLS become wheelchair dependent, although a smaller number of them is able to walk with the use of walking assistive devices. 1 11 12 13

Cognitive deficits range from mild to moderate, and, unlike other neurometabolic diseases, do not progress over the decades. 11 Usually, patients are able to reach a certain level of independency. 1

Other manifestations include preterm birth, pseudobulbar dysarthria, seizures, and ophthalmologic alterations. The latter are present after 3 years of age, with features that are pathognomic when present, characterized by retinal glistening white dots representing crystalline inclusions, and microcysts in the fovea. 1 2 11

Brain imaging plays an important role in the evaluation of patients with SLS, even though there are no pathognomonic findings. Computed tomography (CT) scans depict nonspecific hypodensities in the cerebral white matter. 14 Brain MRI, however, is the imaging modality of choice, due to its lack of ionizing radiation and better characterization of grey and white matter abnormalities, as well as morphological changes. The prominent finding is diffuse, confluent signal alteration in the periventricular and deep white matter sparing the subcortical fibers, reflecting gliosis, delayed myelination, dysmyelination, demyelination, and lipids accumulation. 14 15 A frontal or parieto-occipital predominance has been described. 4 11 Some case studies also report brain atrophy in SLS patients, although there is not sufficient data to conclude if it is progressive. 3 4 16 Magnetic resonance spectroscopy of the white matter abnormality is another useful resource and depicts peaks assigned to lipids in 0.8 to 0.9 ppm and in 1.3 ppm, which probably reflects accumulation of fatty alcohols or their metabolites. 17 18

It is well recognized that the main imaging findings are related with the leukoencephalopathy. Data related to grey matter involvement is scarce, with some neuropathological studies reporting conflicting results concerning neuronal population loss of the putamen, the substantia nigra, and the thalamus. 19 20 21 22 23 A limitation for most of these studies is that there was no definitive confirmation of the SLS diagnosis. Jagel and Jagel et al. 24 25 mention some that presented cases with typical SLS features 19 20 21 and other with atypical features. 22 23 More recently, Staps et al. 26 conducted a postmortem analysis of a genetically confirmed SLS patient. Taking in consideration only the reports with typical clinical features and the one with confirmed diagnosis, there are still conflicting results. Sylvester 19 describes fat droplets in grey matter as well as an increased number of astrocytes in the cortex, caudate, and lentiform nuclei; also noted were a slight loss of Purkinje cells in the cerebellum and focal areas of cerebellar atrophy. Silva et al. 20 reports normal neuronal population and myelination in the cerebral cortex, the basal ganglia, and the internal capsule; the only finding was demyelination of the corticospinal tracts in the medulla. Wester et al., 21 however, analyzed the brain of two SLS patients and reported a slight loss of neurons in the telencephalic cortex, the putamen, and the striatum, with the most striking degeneration in the substantia nigra *.* Finally, Staps et al. 26 found no grey matter neuronal loss but concluded that the disturbed lipid profile affects both white and grey matter, thus suggesting that it is not a pure leukoencephalopathy.

In the present study, using brain volumetry, we were able to noninvasively evaluate and compare deep grey matter, brain stem and cerebellar grey matter volume of SLS patients with a healthy control group. It should be noted that the reduced grey matter areas found in the present study are directly or indirectly involved in motor functions. Clinically, there are motor deteriorations throughout the lives of SLS patient, not accompanied by cognitive deterioration, thus leading to the postulation that they are secondary to limitations during the motor development age. 13 However, with proofing of atrophy of structures related to movement control, it can be questioned whether these deteriorations are not directly caused by the disease itself.

Interestingly, it is well-known that the thalamus is part of pathways between the cerebellum, the basal ganglia, and the frontal cortex. Targeted thalamotomies and deep brain stimulation have been successfully used to treat involuntary movements, confirming its essential role in motor functions. There are cerebellum-recipient thalamus nuclei 27 and basal ganglia-recipient (pallidum and substantia nigra) thalamus nuclei, with afferent pathways to the frontal cortex. 28 Coincidentally, the structures involved in these pathways are the structures with significant volume reduction in the present study (that is, the cerebellum, the thalamus, and the pallidum). The substantia nigra was not evaluated in our analysis, but, as noted before, was shown to be strikingly affected in a previous report. 21 Perhaps, disruption of these pathways is accountable for both the spastic diplegia present in these patients and the volume reduction observed in the present study.

To our knowledge, no article specifically addresses brainstem alterations in SLS patients, but there is evidence of demyelination of corticospinal tracts. 20 In addition to that, we can postulate that cerebellothalamic tracts involvement is also related to the brainstem volume reduction observed in our analysis.

The present study brings light to the possibility that Sjogren-Larsson syndrome has a more pronounced involvement in the thalamic motor pathways. With this new knowledge, more studies could better investigate grey matter alterations in these patients in order to try to establish if these are related to the motor disability clinically observed, whether as the cause of it or as a long-term consequence.

We consider the hypothesis that motor function deterioration might be related to the reduced grey matter areas found in the present study, as these structures are related to movement control; in particular, the present study brings light to the possibility that SLS has a more pronounced involvement in the thalamic motor pathways, while striatal structures would be less interested. Further investigation on a possible selective involvement of grey matter areas or white matter tracts in these patients are needed, maybe integrating DTI studies, which may provide information about white matter lesions. 30

A critical inquiry arises as to whether these deteriorations stem directly from the disease itself or if they represent long-term consequences. The present study carries a limitation rooted in the application of FreeSurfer, which holds the potential to introduce errors, especially within regions of white matter hyperintensity, consequently leading to inaccuracies in the segmentation of gray matter. 29 It is important to acknowledge further limitations within our study, notably the relatively small sample size and the examination of MRIs of the SLS patients at a single time point, involving individuals in their adult years --by which juncture symptoms had already experienced substantial progression. Although reports of cognitive impairment are present, definitive conclusions regarding the evolution of imaging findings remain constrained by the paucity of data. It is crucial to recognize that SLS encompasses a broad spectrum of genotypes and phenotypes, which might account for the seemingly inconsistent data present within the literature.

Data obtained from this automated segmentation software using only a T1-weighted volumetric sequence can noninvasively lead to new understandings of the disease.
